# Comparative Evaluation of Different Extraction Techniques and Solvents for the Assay of Phytochemicals and Antioxidant Activity of Hashemi Rice Bran

**DOI:** 10.3390/molecules200610822

**Published:** 2015-06-11

**Authors:** Ali Ghasemzadeh, Hawa Z. E. Jaafar, Abdul Shukor Juraimi, Amin Tayebi-Meigooni

**Affiliations:** Department of Crop Science, Faculty of Agriculture, Universiti Putra Malaysia, 43400 Serdang, Selangor, Malaysia; E-Mails: hawazej@upm.edu.my (H.Z.E.J.); ashukur@upm.edu.my (A.S.J.); amin_ir@hotmail.com (A.T.-M.)

**Keywords:** Hashemi rice bran, ultra-high performance liquid chromatography, DPPH, nitric oxide scavenging, β-carotene bleaching, ultrasonic

## Abstract

Secondary metabolite contents (total phenolic, flavonoid, tocopherol, and tocotrienol) and antioxidant activities of Hashemi rice bran extracts obtained by ultrasound-assisted and traditional solvent (ethanol and 50:50 (*v*/*v*) ethanol-water) extraction techniques were compared. Phenolic and, flavonoid compounds were identified using ultra-high performance liquid chromatography and method validation was performed. Significant differences (*p* < 0.05) were observed among the different extraction techniques upon comparison of phytochemical contents and antioxidant activities. The extracts obtained using the ethanol-water (50:50 *v*/*v*) ultrasonic technique showed the highest amounts of total phenolics (288.40 mg/100 g dry material (DM)), total flavonoids (156.20 mg/100 g DM), and total tocotrienols (56.23 mg/100 g DM), and the highest antioxidant activity (84.21% 1,1-diphenyl-2-picrylhydrazyl (DPPH), 65.27% β-carotene-linoleic bleaching and 82.20% nitric oxide scavenging activity). Secondary metabolite contents and antioxidant activities of the rice bran extracts varied depending of the extraction method used, and according to their effectiveness, these were organized in a decreasing order as follows: ethanol-water (50:50 *v*/*v*) ultrasonic, ethanol-water (50:50 *v*/*v*) maceration, ethanol ultrasonic and ethanol maceration methods. Ferulic, gallic and chlorogenic acids were the most abundant phenolic compounds in rice bran extracts. The phytochemical constituents of Hashemi rice bran and its antioxidant properties provides insights into its potential application to promote health.

## 1. Introduction

Rice (*Oryza sativa* L.) is the_,_ most important cereal crop in the world and, is the staple food for about half of the world’s population. Like other cereal grains rice is rich in nutrient components such as carbohydrates, proteins, certain fatty acids, and micronutrients (vitamins and trace minerals) [1,2]. In addition, rice is a source of many_,_ bioactive compounds and phytochemicals, known as antioxidants, including phenolic compounds [3,4,5]. During rice milling, rice bran_,_ is produced as a by-product which is reported to be an excellent source of minerals and vitamins [6]. Rice bran has a_,_ high nutritive value and beneficial health effects such as blood cholesterol lowering, laxative effect, and reducing the incidence of atherosclerosis disease [7,8]. Polyphenols are the most important group of phytochemicals [9,10] as they exhibit health-promoting properties, including protective effects against cardiovascular diseases [11], antioxidant properties [12,13], and anti-inflammatory [14] and anticancer activities [15,16]. Blood lipid and glucose reduction, and enhanced human immunity due to intake of flavonoids have been reported in several studies [17,18]. Rice is a good source of phenolic_,_ compounds [19,20]. The phenolic compounds commonly present in whole grains are phenolic acids and flavonoids. In whole grains, gallic, caffeic, ferulic, vanillic, syringic, cinnamic, and protocatechuic acids were reported as common phenolic acids [21]. Gallic [22], ferulic [23], caffeic [24], and cinnamic acids [25] have been reported to be natural antioxidants, which are free radical scavengers and protect the human body from the effectives of oxidative stress. In a recent study, ferulic and *p*-coumaric acids were identified as the most abundant phenolic acids in bran of most rice varieties [26]. Isolation and identification of polyphenols from plants, herbs, and spices among others is mostly dependent on the extraction solvent and technique used. Several extraction techniques have previously been reported in order to extract phenolic_,_ compounds from plant materials such as microwave [27] and ultrasound-assisted methods [28], supercritical fluid extraction methods [29], the shake-flask, technique [29], reflux [30] and Soxhlet extractions [31]. Hashemi rice is a popular rice variety in the north of Iran where most people use this rice for cooking. To the best of our knowledge, there, is little information regarding the phenolic compounds found in Hashemi rice bran and their potential antioxidant activity. Furthermore, extraction and ultra-high performance liquid chromatography (UHPLC) analysis techniques have not been developed for this rice variety. Thus, the aim of this study was to investigate the extraction efficiency of phenolic compounds and flavonoids from Hashemi rice bran, validate the corresponding UHPLC method, and characterize their antioxidant activity.

## 2. Results and Discussion

### 2.1. Total Phenolic and Flavonoid Contents

The results from the present study showed that aqueous ethanol extracts had higher total phenolic (TP) and flavonoid (TF) contents than absolute ethanol extracts. Significant differences (*p* < 0.05) were observed depending on the solvent and extraction technique used for TP and TF contents. As shown in Table 1, ultrasonic rice bran extraction using ethanol-water (50:50 *v*/*v*) gave the highest content of phenolic compounds (288.40 mg/100 g dry material (DM)), followed by the ethanol-water (50:50 *v*/*v*) maceration (270.51 mg/100 g DM), ethanol ultrasonic (246.34 mg/100 g DM), and ethanol maceration (221.06 mg/100 g DM) extractions. The total flavonoid contents of Hashemi rice bran ranged from 156.20 to 108.50 mg/100 g DM. Like the TP content, the highest TF content was observed when the ultrasonic-assisted rice bran extraction using ethanol-water (50:50 *v*/*v*) was used. This may be related to the fact that polyphenols are more soluble in more_,_ polar solvents such as aqueous ethanol than in less polar solvents such as absolute ethanol [32,33]. In addition, the mixture viscosity decreased because of the presence of water, which might have improved the mass transfer. The result of a recent study showed that Hashemi rice bran represented the highest contents of phenolic and flavonoid compounds, with respective values of 3.95 and 0.85 mg/g DM, compared to Ali Kazemi, Neda, Binam and Shirodi varieties [34]. 

**Table 1:** Total phenolic, total flavonoid and total tocopherol content of Hashemi rice bran extract with different extraction technique.

Extraction Solvent/Technique	TPC (mg/100 g DM)	TFC (mg/100 g DM)	Total Tocopherol (mg/100 g DM )	Total Tocotrienols (mg/100 g DM )
Ethanol maceration	221.06 ± 10.63 ^d^	108.50 ± 10.01 ^c^	38.11 ± 2.04 ^a^	46.54 ± 2.92 ^c^
Ethanol-water (50:50) maceration	270.51 ± 11.47 ^b^	137.15 ± 12.89 ^b^	36.93 ± 2.26 ^a^	55.83 ± 1.85 ^a^
Ethanol ultrasonic	246.34 ± 12.26 ^c^	112.60 ± 13.65 ^c^	37.08 ± 2.21 ^a^	51.28 ± 2.80 ^b^
Ethanol-water (50:50) ultrasonic	288.40 ± 14.35 ^a^	156.20 ± 10.69 ^a^	37.51 ± 2.05 ^a^	56.23 ± 2.37 ^a^

TPC: total phenolic content; TFC: total flavonoid content; Data are means of triplicate, measurements ± standard deviation. Means not sharing a common single letter for each measurement were significantly_,_ different at *p* < 0.05.

The use of ultrasonic ethanol-water (50:50 *v*/*v*) extraction, due to the effect of the solvent properties on cavitational bubbles which provide force to collapse plant tissues during rice bran extraction, can result in significant differences in phenolic compound contents compared with other extraction methods. In the extraction method using the ethanol-water (50:50 *v*/*v*) mixture, ethanol (higher vapor pressure) produces more bubbles than water (lower vapor pressure). Moreover, the surface tension of the liquid is another feature that contributes to the formation of cavitational bubbles. In liquids with lower surface tension, cavitational bubbles are created more easily because the ultrasonic energy applied can more easily exceed the surface tension. Thus, the ethanol-water (50:50 *v*/*v*) mixture was more effective in phenolic compound extraction and can apply a greater force to plant tissues. If we consider liquid viscosity, liquids with low viscosity are more effective because the ultrasonic energy can more easily overcome the molecular forces of low viscosity liquids. In addition, low viscosity liquids can also easily penetrate plant tissues due to their low density and high diffusivity. Therefore, in the ultrasonic extraction the ethanol and water mixture can help to extract more phenolic contents from Hashemi rice bran, so ethanol-water (50:50 *v*/*v*) ultrasonic extraction was selected for future experiments for the isolation of phenolic acids and flavonoids.

### 2.2. Total Tocopherol and Tocotrienol Contents

Tocopherol and tocotrienol are commonly known as vitamin E and are the main antioxidants present in rice bran [35]. Tocopherol and tocotrienol constitute a series of related benzopyranols that are produced in plant tissues and are powerful lipid-soluble antioxidants. Considering total tocopherol content, ethanol maceration was the most effective extraction method (38.11 mg/100 g DM) when compared with other extraction methods, including ethanol-water (50:50 *v*/*v*) ultrasonic (37.51 mg/100 g DM), ethanol ultrasonic (37.08 mg/100 g DM), and ethanol-water (50:50 *v*/*v*) maceration (36.93 mg/100 g DM) extractions (Table 1). However, there were no significant differences in total tocopherol contents among the various extraction methods used. A high content of total tocotrienols (56.23 mg/100 g DM) was observed when the ethanol-water (50:50, *v*/*v*) ultrasonic extraction method was used. Significant differences (*p* < 0.05) were observed when the effectivity of the different extraction methods and solvents was assessed regarding tocotrienol extraction. A recent study showed that tocotrienols (specifically γ-tocotrienol) are more abundant in rice bran than tocopherols [36]. In addition, higher vitamin E contents were found in rice bran than in whole rice and milled rice [35,37]. The extraction procedure, especially the extracting solvent, contributes to the amount of phytochemicals that can be recovered from different samples [32,33]. The results of the present study showed that solvent polarity has an important role in the extraction of total tocotrienols. Thus, the more polar solvents, such as the ethanol-water (50:50 *v*/*v*) mixture, extracted more vitamin E compared with absolute ethanol, a result that is consistent with those from previous studies [38,39].

### 2.3. Antioxidant Activity of Hashemi Rice Bran Extracts

#### 2.3.1. 1,1-Diphenyl-2-picrylhydrazyl (DPPH) Radical Scavenging Assay

**Figure 1:**
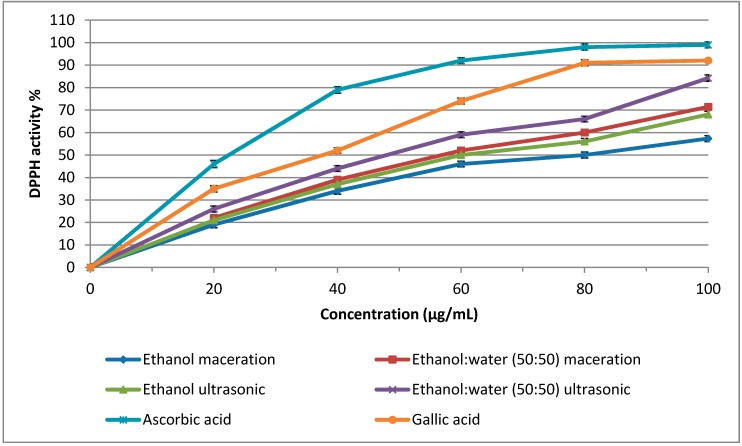
Free radical scaven_._ging activity (DP_._PH assay) of t_._he different extrac_._t of Hashemi rice bran. Error bars represent standard errors of the mean (*n* = 3).

The DPPH radical scavenging activities of Hashemi rice bran extracts obtained through different extraction methods are shown in Table 2 and Figure 1. Significant differences (*p* < 0.05) were observed among the extracts obtained through different extraction methods for DPPH radical scavenging activity. The DPPH radical scavenging activity of the extracts was ranked in the following order: ethanol-water (50:50 *v*/*v*) ultrasonic (84.21%), ethanol-water (50:50 *v*/*v*) maceration (71.41%), ethanol ultrasonic (68.05%), and ethanol maceration (57.33%) methods. No significant difference was observed between the ethanol ultrasonic and the ethanol maceration methods. As shown in Figure 1, a half maximal inhibitory concentration (IC_50_) of 47.0 µg/mL was observed for the ethanol-water (50:50 *v*/*v*) ultrasonic extraction technique. The IC_50_ values for the ethanol ultrasonic, ethanol-water (50:50 *v*/*v*) maceration, and ethanol maceration extraction methods were 56.2, 60.8, and 64.0 µg/mL, respectively. Gallic acid and ascorbic acid, with IC_50_ values of 37.2 and 24.6 µg/mL, respectively, were used as a positive control. As shown in Figure 1, the radical scavenging DPPH activities of the Hashemi rice bran extracts were lower than those of gallic acid and ascorbic acid. In the current study, by using the ultrasonic extraction method and ethanol-water (50:50 *v*/*v*) solvent, the IC_50_ value of Hashemi rice bran (47.0 µg/mL) was improved in comparison to a previous study which reported an IC_50_ value of 169.86 µg/mL using a different extraction technique (reflux) and solvent (methanol) for extraction [34]. Previous studies have reported that the reducing power of phenolic compounds is higher than that of α-tocopherol [40]. Furthermore, phenolic compounds exhibit four times higher antioxidant activities than γ-oryzanol [41] and α-tocopherol [42,43]. γ-oryzanol has also been reported to have a higher antioxidant activity than tocopherols (almost 10 times higher), while tocopherols show antioxidant activities approximately 40–60 times lower than those of tocotrienols [41,44]. In the present study, the highest contents of tocotrienols and phenolic compounds were observed when the ethanol-water (50:50 *v*/*v*) ultrasonic extraction technique was used, and the high antioxidant activity of the Hashemi rice bran extracts obtained with this technique could be related to the high levels of phenolics and tocotrienols. The DPPH radical scavengi_._ng assay is an important method to determine the antioxidant activity of plant extracts, but it cannot provide enough information regarding the antioxidant activities of phenolic extracts in food items. Consequently, the antioxidant activity of the extracts was measured using the β-carotene bleaching assay.

**Table 2:** Effects of extracting solvent/technique on the antioxidant activity of Hashemi rice bran extracts (100 µg/mL), using three different methods.

Extraction Solvent/Technique	DPPH Assay (%)	β-Carotene-linoleic Acid Bleaching Assay (%)	Nitric Oxide Scavenging Activity (%)
Ethanol maceration	57.33 ± 3.51 ^d^	47.23 ± 2.55 ^c^	46.20 ± 3.78 ^d^
Ethanol-water (50:50) maceration	71.41 ± 2.84 ^b^	53.67 ± 1.94 ^b^	74.50 ± 2.56 ^b^
Ethanol ultrasonic	68.05 ± 1.55 ^c^	54.76 ± 3.17 ^b^	58.30 ± 2.44 ^c^
Ethanol-water (50:50) ultrasonic	84.21 ± 3.84 ^a^	65.27 ± 2.73 ^a^	82.20 ± 2.69 ^a^

Data are means of triplicate measurements ± standard deviation. Means not sharing a common single letter for each measurement were significantly_,_ different at *p* < 0.05.

#### 2.3.2. β-Carotene Bleaching Assay

In this method, peroxyl radicals are made by oxidation of linoleic acid and these radicals oxidize unsaturated β-carotene. Thus, if antioxidants are present in the evaluated sample, β-carotene degradation is reduced. Therefore, the amount of decomposed β-carotene is related to the antioxidant activity of the extract [45,46]. The effects on β-carotene oxidation of Hashemi rice bran extracts obtained using different extraction methods are shown in Table 2. It is obvious that extracts can scavenge free radicals from the heterogeneous medium. As shown in Table 2, the ethanol-water (50:50 *v*/*v*) ultrasonic method was the most effective extraction method in preserving the antioxidant activity of rice bran extracts (65.27%) followed by the ethanol ultrasonic (54.76%), ethanol-water (50:50 *v*/*v*) maceration (53.67%), and ethanol maceration (47.23%) methods. Ascorbic acid showed the highest oxidation inhibition (89.16%). Therefore, we focused on the use of the ethanol-water (50:50 *v*/*v*) ultrasonic method to obtain Hashemi rice bran extracts due to the high amounts of total phenolic and tocopherol contents, high radical scavenging activity, and high β-carotene bleaching inhibition. A linear correlation between the antioxidant activity and polyphenolic contents has been reported as variable ranges in different plants [47,48,49]. Arab *et al.* [50] reported that Fajr rice bran with high total phenolic content showed high antioxidant activity measured using the DPPH radical scavenging assay.

#### 2.3.3. Nitric Oxide Scavenging Activity

**Figure 2:**
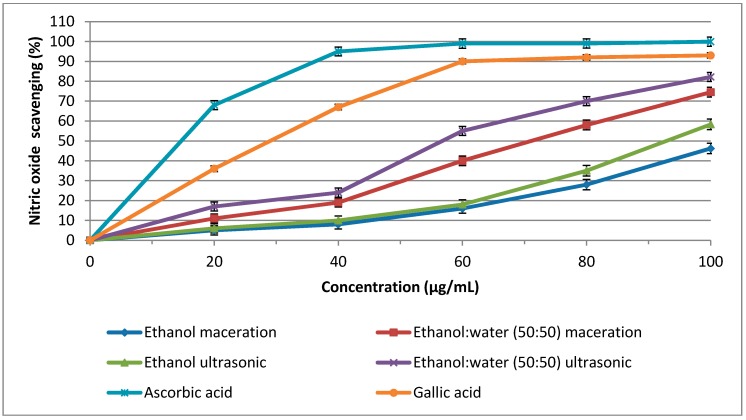
Nitric oxide scavengi_._ng activity of Hashemi rice bran_,_ extracts from different extraction methods. Error bars represent standard errors of the mean (*n* = 3).

As shown in Table 2 and Figure 2, the highest nitric oxide scavenging activity (82.20 μg/mL) with an IC_50_ value of 58.25 μg/mL was observed when the ethanol-water (50:50 *v*/*v*) ultrasonic extraction technique was used. The IC_50_ values of the ethanol ultrasonic, ethanol-water (50:50 *v*/*v*) maceration, and ethanol maceration extraction methods were 70.15, 93.20, and 112.85 μg/mL, respectively (Figure 2). Gallic acid and ascorbic acid showed an IC_50_ value of 27.5 and 15.90 μg/mL, respectively. Excess nitric oxide, which is known to accumulate in the acidic environment of the stomach, reacts with oxygen to form nitrite ions and induce mutagenic reactions [51]. Thus, the nitric oxide produced must be scavenged from the human body and previous studies have shown that phenolic compounds have a great nitrite scavenging activity [52]. In the present study, we used three different antioxidant assays and the results of all three assays showed that the ethanol-water (50:50 *v*/*v*) ultrasonic extraction method resulted in the highest antioxidant activity. In addition, secondary metabolites analysis showed that the ethanol-water (50:50 *v*/*v*) ultrasonic extraction method produced the highest TP, TF, and total tocotrienols content values. Phenolic acids and flavonoids are phytochemicals and act as potent antioxidant agents. Consequently, the highest antioxidant activity recorded in extracts obtained using the ethanol: water (50:50 *v*/*v*) ultrasonic extraction method might be related to high secondary metabolite contents. A correlation analysis was carried out in order to test this hypothesis.

### 2.4. Correlation Analysis 

Table 3 shows the intercorrelations among the different measurements carried out in Hashemi rice bran extracts. A significant (*p* < 0.01) positive correlation was observed between TP, TF, DPPH, and nitric oxide scavenging activities. In addition, a positive and significant (*p* < 0.01) correlation was found between total tocotrienols, DPPH, and nitric oxide scavenging activities. 

**Table 3:** Correlatio_._n analysis betw_._een_,_ secondary metabolites and antioxi_._dant activity of Hashemi rice bran.

		1	2	3	4	5	6	7
1	TP	1						
2	TF	0.825 **	1					
3	Total tocopherol	0.526	0.663	1				
4	Total tocotrienols	0.792 *	0.677	0.546	1			
5	DPPH	0.925 **	0.911 **	0.946 **	0.988 **	1		
6	β-Carotene-linoleic acid bleaching	0.668	0.871 *	0.719	0.844 *	0.612	1	
7	Nitric oxide scavenging activity	0.950 **	0.936 **	0.812 *	0.969 **	0.905 **	0.746	1

* = significant at *p* < 0.05; ** = significant at *p* < 0.01.

In the present study, no significant correlation (*p* > 0.05) was found between β-carotene bleaching assay, TP, and total tocopherol content. The β-carotene bleaching assay only showed a significant correlation (*p* < 0.05) with TF and total tocotrienols. Correlation coefficients and regression analyses showed that phenolic compounds, tocopherols, and tocotrienols were responsible for the antioxidant activity in the Hashemi rice bran extracts. A positive and significant correlation, between polyphenols and antioxidant activity has already been reported in several previous studies [9,28,53,54].

### 2.5. Phenolic Acid and Flavonoid Composition of Hashemi Rice Bran Extracts

Extracts obtained using the ethanol-water (50:50, *v*/*v*) ultrasonic method were chosen for phenolic and flavonoid profiling because they showed the highest phenolic and flavonoid contents when compared with other extraction methods. As shown in Table 4, Hashemi rice bran is a good, source of phenolic compounds. 

**Table 4:** Concentration of identified phenolic acids and flavonoids and their recovery test in Hashemi rice bran extract.

Phenolic Acids	Concentration in Sample (mg/100 g DM)	Standard Added (mg)	Recovery		Recovery (%)	RSD (%)
			Expected	Actual		
Gallic acid	11.56 ± 0.88	1.00	12.56	12.16 ± 0.26	96.81	2.13
	11.56 ± 0.88	2.50	14.06	13.68 ± 0.37	97.30	2.70
	11.56 ± 0.88	5.00	16.56	16.24 ± 0.24	98.07	1.47
Protocatechuic acid	6.72 ± 0.16	1.00	7.72	8.80 ± 0.10	113.99	1.13
	6.72 ± 0.16	2.50	9.22	10.12 ± 0.15	109.76	1.48
	6.72 ± 0.16	5.00	11.72	11.95 ± 0.13	101.96	1.08
Syringic acid	10.39 ± 0.11	1.00	11.39	10.55 ± 0.075	92.63	0.71
	10.39 ± 0.11	2.50	12.89	11.47 ± 0.03	88.98	0.26
	10.39 ± 0.11	5.00	15.39	16.42 ± 0.09	106.69	0.54
Chlorogenic acid	11.12 ± 0.28	1.00	12.12	11.80 ± 0.21	97.36	1.77
	11.12 ± 0.28	2.50	13.52	14.19 ± 0.18	104.96	1.26
	11.12 ± 0.28	5.00	16.12	15.33 ± 0.17	95.10	1.10
Caffeic acid	10.59 ± 0.16	1.00	11.59	10.64 ± 0.09	91.80	0.84
	10.59 ± 0.16	2.50	13.09	12.77 ± 0.11	97.56	0.86
	10.59 ± 0.16	5.00	15.59	16.36 ± 0.07	104.94	0.42
Ferulic acid	12.28 ± 0.69	1.00	13.28	12.91 ± 0.16	97.21	1.23
	12.28 ± 0.69	2.50	14.78	15.10 ± 0.19	102.17	1.25
	12.28 ± 0.69	5.00	17.28	16.52 ± 0.23	95.60	1.39
Cinnamic acid	8.23 ± 0.86	1.00	9.23	10.66 ± 0.14	115.49	1.31
	8.23 ± 0.86	2.50	10.73	11.91 ± 0.17	111.00	1.42
	8.23 ± 0.86	5.00	13.23	12.46 ± 0.18	94.18	1.44
Apigenin	2.65 ± 0.52	0.25	2.90	3.35 ± 0.12	115.51	3.58
	2.65 ± 0.52	0.50	3.15	3.60 ± 0.09	114.28	2.50
	2.65 ± 0.52	1.00	3.65	3.40 ± 0.06	93.15	1.76
Catechin	4.28 ± 0.88	0.50	4.78	4.20 ± 0.10	87.86	2.38
	4.28 ± 0.88	1.00	5.28	5.00 ± 0.11	95.23	2.20
	4.28 ± 0.88	2.00	6.28	5.85 ± 0.13	93.15	2.22
Quercetin	1.36 ± 0.22	0.25	1.61	1.50 ± 0.01	93.16	0.66
	1.36 ± 0.29	0.50	1.86	1.62 ± 0.03	87.09	1.85
	1.36 ± 0.18	1.00	2.36	2.46 ± 0.04	104.23	1.62

In the present study, seven phenolic acids (gallic, protocatechuic, syringic, chlorogenic, caffeic, ferulic, and cinnamic acids) and three flavonoids (apigenin, catechin, and quercetin) were detected in the extracts. The results showed that ferulic acid (12.28 mg/100 g D.W), gallic acid (11.56 mg/100 g DM), and chlorogenic acid (11.12 mg/100 g DM) were the most abundant phenolic acids within the identified compounds. Three flavonoid compounds, namely apigenin, catechin, and quercetin, were identified in rice bran extracts at concentrations of 2.65, 4.28, and 1.36 mg/100 g DM, respectively. The rest of the phenolic compounds were present in the following order of decreasing concentration: ferulic, gallic, chlorogenic, caffeic, syringic, and cinnamic acids. Recently other flavonoids have been identified in other rice varieties, including naringenin [55], luteolin, apigenin [56], rutin [57], and myricetin [58].

### 2.6. Validation Method

A recovery study, of extracts from rice bran was carried out, by adding different spike levels (low, medium, and high) of phenolic and flavonoid standards in order to evaluate, the accuracy of the analytical methods. The mean recovery was 97.30%–105.19%, 101.96%–113.99%, 88.98%–106.69%, 95.10%–104.96%, 91.80%–104.94%, 95.60%–102.17%, 94.18%–115.49%, 93.15%–115.51%, and 87.86%%–95.23% for gallic, protocatechuic, syringic, chlorogenic, caffeic, ferulic, cinnamic acids, and for apigenin, and catechin, respectively. The %RSD of the average recovery was 2.11%, 1.24%, 0.51%, 1.39%, 0.90%, 1.30%, 1.40%, 2.61%, and 2.26% for gallic, protocatechuic, syringic, chlorogenic, caffeic, ferulic, cinnamic acids, and for apigenin, and catechin, respectively. The low RSD values indicated that UHPLC system was suitable. The obsorved variation confirmed the robustness of the analysis system. A calibration curve was constructed to assess the linearity between the six concentrations of each phenolic compound and the corresponding peak area of the UHPLC methods. The limit of detection (LOD) is usually defined as the lowest quantity or concentration of a component that can be reliably detected with a given analytical method, but do not have to quantitate as an appropriate value. The limit of quantification (LOQ) is usually defined as the lowest sample concentration which can still be quantitatively detected with accuracy and an acceptable precision. In current study, LOQ, and LOD ranged from 0.2–0.4 (µg/mL) and 0.01–0.5, respectively.

**Table 5:** Analytical characteristics for determination of phenolic compounds.

Compounds	Regression Equation (y = ax ± b)	R^2^	LOD (µg/mL)	LOQ (µg/mL)
Gallic acid	y = 1846.5x − 5.2530	0.9954	0.50	0.25
Protocatechuic acid	y = 2348.1x + 14.863	0.9990	0.10	0.30
Syringic acid	y = 2245.2x − 3.5633	0.9992	0.10	0.30
Chlorogenic acid	y = 2988.4x − 29.643	0.9984	0.01	0.20
Caffeic acid	y = 3357.1x + 8.7350	0.9990	0.13	0.40
Ferulic acid	y = 1969.5x − 14.299	0.9980	0.01	0.20
Cinnamic acid	y = 3144.8x + 21.206	0.9982	0.03	0.20
Apigenin	y = 1654.7x − 22.30	0.9975	1.07	3.25
Catechin	y = 2459.4x + 71.083	0.9990	0.85	2.58
Quercetin	y = 14692x + 2972.80	0.9981	0.19	0.49

y = peak area; R^2^ = coefficient of determination; LOD: Limit of detection; LOQ: Limit of quantification.

## 3. Experimental Section 

### 3.1. Preparation of Hashemi Rice Bran Extracts

Hashemi rice variety was donated by the Rice Research Institute of Iran (RRII). All paddy rice samples were dehulled and polished using rice dehusker and rice milling machine, set at 8% degree of milling, to obtain the milled rice bran and then, in order to separate the grains from the rice bran, they were sieved through 180 μm sieve (80 mesh). In order to inactivate endogenous lipases, rice bran was heated at 100 °C for 15 min. Rice bran powder (100 gr) was extracted_,_ with 1 L ethanol and ethanol-water (50:50 *v*/*v*) for 1 h in a shaking water bath set at 160 rpm and 45 °C. The solvents were evaporated after filtering through a Whatman No. 1 filter paper. The remaining solid residues of rice bran were extracted twice more using a similar procedure and the extracts were mixed, before removing the solvent using a vacuum oven. For the ultrasonic extraction, the temperature was set at 45 °C and the ultrasonic power was adjusted to 150 W. Samples were extracted for 1 h. All extracts were_,_ stored at −20 °C for future analysis. 

### 3.2. Total Phenolic Content

Hashemi rice bran extracts (100 µL) were diluted in 10 mL of distilled_,_ water. Folin-Ciocalteu reagent (500 µL) was added to this solution and incubated in total darkness for 10 min at room temperature. Later, sodium carbonate 20% (1 mL) was added and solutions were incubated again for 20 min. After incubation, the absorbance of the solutions was read at 760 nm using a spectrophotometer (UV_._2550, Shimadzu, Kyoto, Japan). Gallic acid_,_ standard at different conc_e_ntrations was used for the calibration curve calculation [59].

### 3.3. Total Flavonoid Content

Hashemi rice bran extracts (1 mL) were mixed with NaNO_2_ in a methanolic solution (4 mL, 1:5, *w*/*v*) and kept at room temperature for 6 min. Thereafter, AlCl_3_ solution_,_ (0.3 mL, 1:10, *w*/*v*) was added, mixed well, and allowed to stand for another 6 min. Immediately thereafter, 1 M NaOH solution (2.0 mL) was added to each extract and incubated for 10 min at room temperature. The absorbance of the solutions was read at 510 nm using a spectrophotometer_,_ (UV2550, Shimadzu, Kyoto, Japan). Quercetin standards of different concentrations was used for the calibration curve calculation [60]. 

### 3.4. Total Tocopherol Content 

Extracts (1 mL) were transferred_,_ to a flask, and toluene (5 mL) and 2.2′-bipyridine (3.5 mL, 0.07% *w*/*v* in 95% ethanol) were added to the mixture. Next, FeCl_3_.6H_2_O (0.5 mL, 0.2% *w*/*v* in 95% ethanol) was added and solutions were kept for 1 min. The absorbance of the solutions was read a_._t 520 nm using a spectrophotometer (UV2550, Shimadzu, Kyoto, Japan). During their preparation, all solutions were kept away from the light. α-tocopherol standard was dissolved in toluene in a concentration_,_ range of 0–320 μg/mL and the calibration curve was calculated. The total tocopherol amount was calculated based on μg of α-tocopherol per mL of extract [61].

### 3.5. In Vitro Evaluation of Antioxidant Activity

#### 3.5.1. 1,1-Diphenyl-2-picrylhydrazyl (DPPH) Assay

The DPPH assay was used in order to evaluate the free radical scavenging activity of Hashemi rice bran extracts [62]. DPPH was dissolved_,_ in methanol at a concentration of 200 µM. The DPPH solution (2 mL) was mixed with Hashemi rice bran extract (2 mL) and incubated in a dark room for 30 min at 28 °C. After incubation, the absorbance of the samples was read at 517 nm using a spectrophotometer (UV_._2550, Shimadzu, Kyoto, Japan). Gallic acid and ascorbic ac_._id were used as a positive control. The scavenging activity was calculated using the following formula:

% inhibition = [(absorbance_control_ − absorbance_sample_)/absorbance_control_)] × 100 (1)

#### 3.5.2. β-Carotene Bleaching Assay

Lipid peroxidation_,_ inhibition activity of Hashemi rice bran extracts was determined using the β-carotene bleaching method [63]. β-Carotene (5 mg) was dissolved in HPLC-grade chloroform (10 mL). Tween 40 (400 mg) was mixed with linoleic acid (40 mg); then the β-carotene solution (600 μL) was added and mixed gently. Chloroform was evaporated from the solution using a rotary evaporator (Cole-Parmer Diagonal Rotary_,_ Evaporator System, Chicago, IL, USA). The residue was dissolved in distilled water (100 mL) and an aliquot of this solution (2.5 mL) was transferred to a test tube. Hashemi rice bran extract (350 μL, 2 g/L) was added. After this, samples were heated in a water bath for 120 min at 50 °C. The absorbance values of the samples were read spectrophotometrically at 470 nm. The antioxidant capacity_,_ of the extracts was expressed as percentage inhibition:

% inhibition = [(absorbance_control_ − absorbance_sample_)/absorbance_control_)] × 100 (2)

#### 3.5.3. Nitric Oxide Scavenging Activity

Hashemi rice bran extract (3 mL) at different concentrations (50–250 μg/mL) was transferred to the test tubes. Thereafter, 2 mL of the reaction mixture [10 mM sodium nitroprusside (SNP) in 0.5 M phosphate buffer, pH 7.4] were added and mixed well. The mixture was incubated for 60 min at 37 °C. After incubation, Griess reagent (0.1% α-naphthyl-ethylenediamine in water and 1% H_2_SO_4_ in 5% H_3_PO_4_) was added to the mixtures. The absorbance of the samples was measured spectrophotometrically_,_ at 540 nm (UV2550, Shimadzu, Kyoto, Japan). Gallic acid and ascorbic acid were used as a positive control. Nitric oxide (NO) scavenging activity (%) was calculated by_,_ using the formula:

% NO scavenging activity = [(absorbance_control_ − absorbance_sample_)/absorbance_control_)] × 100 (3)

### 3.6. Separation and Analysis of Flavonoids and Phenolic Acids

Ultra-high performance liquid chromatography (UHPLC, 1290 Infinity Quaternary_,_ LC System, Agilent, Santa Clara, CA, USA) was used for flavonoid separation and identification. The chromatographic system conditions were set as follows: mobile phase, 0.03 M orthophosphoric acid (A) and HPLC grade methanol (B); detector, UV 360 nm; column, C18 column (5.0 µm, 4.6 mm inner diameter [ID] × 250 mm); column oven temperature, 35 °C; and flow rate, 1.0 mL/min. Gradient elution was performed as follows: 0 min 40% B, 10 min_,_ 100% B, 15 min 100% B, and 20 min 40% B. To prepare the standard solution, gallic acid monohydrate (CAS Number 5995-86-8), chlorogenic acid (CAS Number 327-97-9), protocatechuic acid (CAS Number 99-50-3), syringic acid (CAS Number 530-57-4), caffeic acid (CAS Number 331-39-5), ferulic acid (CAS Number 537-98-4), *trance*-cinnamic acid (CAS Number 140-10-3), (+)-catechin hydrate (CAS Number 225937-10-0), apigenin (CAS Number 520-36-5) and quercetin dihydrate (CAS Number 6151-25-3) were dissolved in methanol (HPLC grade) at various concentrations. Linear_,_ regression equations were calculated using the expression Y = aX ± b, where X was the concentration of the related compound and Y the peak area of the compound obtained from UHPLC. The linearity was established by the coefficient, of determination (R^2^) [64]. 

### 3.7. Recovery Test 

A recovery study was carried out in order to test the accuracy of the method. Known amounts of phenolic acid and flavonoid standards at three different concentrations were added to rice bran extracts. The mixtures were injected into UHPLC and the percentage recovery of each phenolic compound from the spiked samples was calculated as follow:

% Recovery = (amount of phenolic acids or flavonoids after spiking × 100)/(original concentration of phenolic acid or flavonoids + spiked amount)
(4)

Percent relative standard deviation (%RSD) = (standard deviation of phenolic acid or flavonoids × 100)/(average content of phenolic acid or flavonoids)
(5)

### 3.8. Statistical Analysis

All data from the study were shown as mean ± SD of three replicates, and means were compared using analysis of variance_,_ (ANOVA) using the Statistical Analysis System software (SAS 9.0, SAS Institute, Cary, NC, USA). The data obtained were manipulated_,_ to calculate statistical values such as means and standard deviations (SD) using Microsoft Excel (Microsoft Inc., Redmond, WA, USA). Group means were compared using Duncan’s tests. The differences were considered significant at *p* < 0.05.

## 4. Conclusions

In this study, a simple and reliable extraction technique, and a validated UHPLC method were developed for the simultaneous separation and quantification of phenolic acids and flavonoids in Hashemi rice bran. In general, the aqueous organic solvent ethanol-water (50:50 *v*/*v*) ultrasonic extraction technique gave the greatest secondary metabolites content and also showed valuable antioxidant activity which was assessed using three different methods. Ferulic, gallic and chlorogenic acids were the most abundant phenolic compounds in Hashemi rice bran. Hashemi rice bran with its large amounts of potent phenolic compounds has good levels of antioxidant activity and the correlation analysis showed that the antioxidant activity in Hashemi rice bran extracts depended on its secondary metabolite contents, especially of phenolic and tocotrienol compounds. Characterization of the phytochemical profile and antioxidant activity of Hashemi rice bran provides insights into its potential application to promote health. Further investigation and experimentation into optimization of the ultrasonic extraction technique with ethanol-water (50:50 *v*/*v*) in order to find best range of variables (liquid to solid ratio, temperature, extraction time) is strongly recommended.

## References

[1] Deepa G., Singh V., Naidu K.A. (2008). Nutrient composition and physicochemical properties of Indian medicinal rice Njavara. Food Chem..

[2] Kadan R., Phillippy B. (2007). Effects of yeast and bran on phytate degradation and minerals in rice bread. J. Food Sci..

[3] Tian S., Nakamura K., Kayahara H. (2004). Analysis of phenolic compounds in white rice, brown rice, and germinated brown rice. J. Agric. Food Chem..

[4] Vichapong J., Sookserm M., Srijesdaruk V., Swatsitang P., Srijaranai S. (2010). High performance liquid chromatographic analysis of phenolic compounds and their antioxidant activities in rice varieties. LWT-Food Sci. Technol..

[5] Tian S., Nakamura K., Cui T., Kayahara H. (2005). High-performance liquid chromatographic determination of phenolic compounds in rice. J. Chromatogr. A.

[6] Zullaikah S., Lai C.C., Vali S.R., Ju Y.H. (2005). A two-step acid-catalyzed process for the production of biodiesel from rice bran oil. Bioresour. Technol..

[7] Qureshi A.A., Salser W.A., Parmar R., Emeson E.E. (2001). Novel tocotrienols of rice bran inhibit atherosclerotic lesions in C57BL/6 ApoE-deficient mice. J. Nutr..

[8] Ling W.H., Cheng Q.X., Ma J., Wang T. (2001). Red and black rice decrease atherosclerotic plaque formation and increase antioxidant status in rabbits. J. Nutr..

[9] Cai Y., Luo Q., Sun M., Corke H. (2004). Antioxidant activity and phenolic compounds of 112 traditional Chinese medicinal plants associated with anticancer. Life Sci..

[10] Ghasemzadeh A., Ghasemzadeh N. (2011). Flavonoids and phenolic acids: Role and biochemical activity in plants and human. J. Med. Plants Res..

[11] Morton L.W., Caccetta R.A.A., Puddey I.B., Croft K.D. (2000). Chemistry and biological effects of dietary phenolic compounds: Relevance to cardiovascular disease. Clin. Exp. Pharmacol. Physiol..

[12] Ghasemzadeh A., Jaafar H.Z., Rahmat A. (2010). Elevated carbon dioxide increases contents of flavonoids and phenolic compounds, and antioxidant activities in Malaysian young ginger (*Zingiber officinale* Roscoe.) varieties. Molecules.

[13] Balasundram N., Sundram K., Samman S. (2006). Phenolic compounds in plants and agri-industrial by-products: Antioxidant activity, occurrence, and potential uses. Food Chem..

[14] Dos Santos M.D., Almeida M.C., Lopes N.P., de Souza G. E. (2006). Evaluation of the anti-inflammatory, analgesic and antipyretic activities of the natural polyphenol chlorogenic acid. Biol. Pharm. Bull..

[15] Ghasemzadeh A., Jaafar H.Z., Karimi E. (2012). Involvement of salicylic acid on antioxidant and anticancer properties, anthocyanin production and chalcone synthase activity in ginger (*Zingiber officinale* Roscoe) varieties. Int. J. Mol. Sci..

[16] Dai J., Mumper R.J. (2010). Plant phenolics: Extraction, analysis and their antioxidant and anticancer properties. Molecules.

[17] Jung U.J., Lee M.K., Park Y.B., Kang M., Choi M.S. (2006). Effect of citrus flavonoids on lipid metabolism and glucose-regulating enzyme mRNA levels in type-2 diabetic mice. Int. J. Biochem. Cell Biol..

[18] Shen Y.C., Chen S.L., Wang C.K. (2007). Contribution of tomato phenolics to antioxidation and down-regulation of blood lipids. J. Agric. Food Chem..

[19] Goufo P., Trindade H. (2014). Rice antioxidants: Phenolic acids, flavonoids, anthocyanins, proanthocyanidins, tocopherols, tocotrienols, Y-oryzanol, and phytic acid. Food Sci. Nutr..

[20] Walter M., Marchesan E. (2011). Phenolic compounds and antioxidant activity of rice. Braz. Arch. Biol. Technol..

[21] Sosulski F., Krygier K., Hogge L. (1982). Free, esterified, and insoluble-bound phenolic acids. 3. Composition of phenolic acids in cereal and potato flours. J. Agric. Food Chem..

[22] Li L., Ng T., Gao W., Li W., Fu M., Niu S., Zhao L., Chen R., Liu F. (2005). Antioxidant activity of gallic acid from rose flowers in senescence accelerated mice. Life Sci..

[23] Kikuzaki H., Hisamoto M., Hirose K., Akiyama K., Taniguchi H. (2002). Antioxidant properties of ferulic acid and its related compounds. J. Agric. Food Chem..

[24] Gulci I. (2006). Antioxidant activity of caffeic acid (3,4-dihydroxycinnamic acid). Toxicology.

[25] Mansouri A., Makris D.P., Kefalas P. (2005). Determination of hydrogen peroxide scavenging activity of cinnamic and benzoic acids employing a highly sensitive peroxyoxalate chemiluminescence-based assay: Structure activity relationships. J. Pharm. Biomed. Anal..

[26] Tuncel N.B., Yilmaz N. (2011). Gamma-oryzanol content, phenolic acid profiles and antioxidant activity of rice milling fractions. Eur. Food Res. Technol..

[27] Proestos C., Komaitis M. (2008). Application of microwave-assisted extraction to the fast extraction of plant phenolic compounds. LWT-Food Sci. Technol..

[28] Ghasemzadeh A., Jaafar H.Z., Karimi E., Rahmat A. (2014). Optimization of ultrasound-assisted extraction of flavonoid compounds and their pharmaceutical activity from curry leaf (*Murraya koenigii* L.) using response surface methodology. BMC Complement. Altern. Med..

[29] Madhava Naidu M., Sulochanamma G., Sampathu S., Srinivas P. (2008). Studies on extraction and antioxidant potential of green coffee. Food Chem..

[30] Ghasemzadeh A., Jaafar H.Z. (2014). Optimization of Reflux Conditions for Total Flavonoid and Total Phenolic Extraction and Enhanced Antioxidant Capacity in Pandan (*Pandanus amaryllifolius* Roxb.) Using Response Surface Methodology. Sci. World J..

[31] Bicchi C., Binello A., Rubiolo P. (2000). Determination of phenolic diterpene antioxidants in rosemary (*Rosmarinus officinalis* L.) with different methods of extraction and analysis. Phytochem. Anal..

[32] Sultana B., Anwar F., Przybylski R. (2007). Antioxidant activity of phenolic components present in barks of *Azadirachta indica*, *Terminalia arjuna*, *Acacia nilotica*, and *Eugenia jambolana* Lam. trees. Food Chem..

[33] Siddhuraju P., Becker K. (2003). Antioxidant properties of various solvent extracts of total phenolic constituents from three different agroclimatic origins of drumstick tree (*Moringa oleifera* Lam.) leaves. J. Agric. Food Chem..

[34] Karimi E., Mehrabanjoubani P., Keshavarzian M., Oskoueian E., Jaafar H.Z., Abdolzadeh A. (2014). Identification and quantification of phenolic and flavonoid components in straw and seed husk of some rice varieties (*Oryza sativa* L.) and their antioxidant properties. J. Sci. Food Agric..

[35] Shammugasamy B., Ramakrishnan Y., Ghazali H.M., Muhammad K. (2014). Tocopherol and tocotrienol contents of different varieties of rice in Malaysia. J. Sci. Food Agric..

[36] Sarmento C., Ferreira S., Hense H. (2006). Supercritical fluid extraction (SFE) of rice bran oil to obtain fractions enriched with tocopherols and tocotrienols. Braz. J. Chem. Eng..

[37] Huang S.H., Ng L.T. (2011). Quantification of tocopherols, tocotrienols, and Y-oryzanol contents and their distribution in some commercial rice varieties in Taiwan. J. Agric. Food Chem..

[38] Hu W., Wells J.H., Shin T.S., Godber J.S. (1996). Comparison of isopropanol and hexane for extraction of vitamin E and oryzanols from stabilized rice bran. J. Am. Oil Chem. Soc..

[39] Xu Z., Godber J.S. (2000). Comparison of supercritical fluid and solvent extraction methods in extracting Î³-oryzanol from rice bran. J. Am. Oil Chem. Soc..

[40] Laokuldilok T., Shoemaker C.F., Jongkaewwattana S., Tulyathan V. (2010). Antioxidants and antioxidant activity of several pigmented rice brans. J. Agric. Food Chem..

[41] Xu Z., Hua N., Godber J.S. (2001). Antioxidant activity of tocopherols, tocotrienols, and γ-oryzanol components from rice bran against cholesterol oxidation accelerated by 2,2′-azobis (2-methylpropionamidine) dihydrochloride. J. Agric. Food Chem..

[42] Kim J.S. (2005). Radical scavenging capacity and antioxidant activity of the E vitamin fraction in rice bran. J. Food Sci..

[43] Goffman F., Bergman C. (2004). Rice kernel phenolic content and its relationship with antiradical efficiency. J. Sci. Food Agric..

[44] Deepam L.A., Sundaresan A., Arumughan C. (2011). Stability of rice bran oil in terms of oryzanol, tocopherols, tocotrienols and sterols. J. Am. Oil Chem. Soc..

[45] Oke F., Aslim B., Ozturk S., Altundag S. (2009). Essential oil composition, antimicrobial and antioxidant activities of *Satureja cuneifolia* Ten. Food Chem..

[46] Sahreen S., Khan M.R., Khan R.A. (2010). Evaluation of antioxidant activities of various solvent extracts of *Carissa opaca* fruits. Food Chem..

[47] Lee K.W., Kim Y.J., Lee H.J., Lee C.Y. (2003). Cocoa has more phenolic phytochemicals and a higher antioxidant capacity than teas and red wine. J. Agric. Food Chem..

[48] Pietta P.G. (2000). Flavonoids as antioxidants. J. Nat. Prod..

[49] Kim D.O., Jeong S.W., Lee C.Y. (2003). Antioxidant capacity of phenolic phytochemicals from various cultivars of plums. Food Chem..

[50] Arab F., Alemzadeh I., Maghsoudi V. (2011). Determination of antioxidant component and activity of rice bran extract. Sci. Iran..

[51] Yin J., Kwon G.J., Wang M.H. (2007). The antioxidant and cytotoxic activities of Sonchus oleraceus L. extracts. Nutr. Res. Pract..

[52] Tundis R., Bonesi M., Deguin B., Loizzo M.R., Menichini F., Conforti F., Tillequin F.O., Menichini F. (2009). Cytotoxic activity and inhibitory effect on nitric oxide production of triterpene saponins from the roots of *Physospermum verticillatum* (Waldst & Kit) (Apiaceae). Bioorg. Med. Chem..

[53] Rao A.S., Reddy S.G., Babu P.P., Reddy A.R. (2010). The antioxidant and antiproliferative activities of methanolic extracts from Njavara rice bran. BMC Complement. Altern. Med..

[54] Djeridane A., Yousfi M., Nadjemi B., Boutassouna D., Stocker P., Vidal N. (2006). Antioxidant activity of some Algerian medicinal plants extracts containing phenolic compounds. Food Chem..

[55] Chen M.H., Choi S.H., Kozukue N., Kim H.J., Friedman M. (2012). Growth-inhibitory effects of pigmented rice bran extracts and three red bran fractions against human cancer cells: relationships with composition and antioxidative activities. J. Agric. Food Chem..

[56] Goufo P., Falco V., Brites C.M., Wessel D.F., Kratz S., Rosa E.A., Carranca C., Trindade H. (2014). Effect of Elevated Carbon Dioxide Concentration on Rice Quality: Nutritive Value, Color, Milling and Cooking/Eating Qualities. Cereal Chem..

[57] Irakli M.N., Samanidou V.F., Biliaderis C.G., Papadoyannis I.N. (2012). Simultaneous determination of phenolic acids and flavonoids in rice using solid-phase extraction and RP-HPLC with photodiode array detection. J. Sep. Sci..

[58] Sriseadka T., Wongpornchai S., Rayanakorn M. (2012). Quantification of flavonoids in black rice by liquid chromatography-negative electrospray ionization tandem mass spectrometry. J. Agric. Food Chem..

[59] Jayaprakasha G., Patil B.S. (2007). *In vitro* evaluation of the antioxidant activities in fruit extracts from citron and blood orange. Food Chem..

[60] Zhishen J., Mengcheng T., Jianming W. (1999). The determination of flavonoid contents in mulberry and their scavenging effects on superoxide radicals. Food Chem..

[61] Wong M., Timms R., Goh E. (1988). Colorimetric determination of total tocopherols in palm oil, olein and stearin. J. Am. Oil Chem. Soc..

[62] Hsu C.F., Zhang L., Peng H., Travas-Sejdic J., Kilmartin P.A. (2008). Scavenging of DPPH free radicals by polypyrrole powders of varying levels of overoxidation and/or reduction. Synth. Met..

[63] Barros L., Ferreira M.J., Queiros B., Ferreira I.C., Baptista P. (2007). Total phenols, ascorbic acid, beta-carotene and lycopene in Portuguese wild edible mushrooms and their antioxidant activities. Food Chem..

[64] Ghasemzadeh A., Nasiri A., Jaafar H.Z., Baghdadi A., Ahmad I. (2014). Changes in Phytochemical Synthesis, Chalcone Synthase Activity and Pharmaceutical Qualities of Sabah Snake Grass (*Clinacanthus nutans* L.) in Relation to Plant Age. Molecules.

